# Intrauterine administration of recombinant human chorionic gonadotropin before embryo transfer on outcome of in vitro fertilization/ intracytoplasmic sperm injection: A randomized clinical trial 

**Published:** 2014-01

**Authors:** Afsoon Zarei, Mohammad Ebrahim Parsanezhad, Masoumeh Younesi, Saeed Alborzi, Jaleh Zolghadri, Alamtaj Samsami, Sedigheh Amooee, Shahintaj Aramesh

**Affiliations:** 1 *Infertility Research Center, Shiraz University of Medical Sciences, Shiraz, Iran.*; 2 *Department of Obstetrics and Gynecology, Shiraz University of Medical Sciences, Shiraz, Iran.*; 3 *Student Research Committee, Shiraz University of Medical Sciences, Shiraz, Iran.*

**Keywords:** *Recombinant human chorionic gonadotropin (rhCG)*, *Intracytoplasmic sperm injection (ICSI)*, *In vitro fertilization (IVF)*, *Implantation rate*, *Pregnancy rate*

## Abstract

**Background:** The direct effect of hCG on the human endometrium was studied several times.

**Objective:** The objectives of this study were to evaluate the effectiveness of intrauterine injection of recombinant human chorionic gonadotropin (rhCG) before embryo transfer (ET).

**Materials and Methods:** In this randomized placebo-controlled clinical trial, a total number of 182 infertile patients undergoing their first in vitro fertilization/ intracytoplasmic sperm injection (IVF-ICSI) cycles were randomly assigned to receive 250μg intrauterine rhCG (n=84) or placebo (n=98) before ET. The implantation and pregnancy rates were compared between groups.

**Results:** Patients who received intrauterine rhCG before ET had significantly higher implantation (36.9% vs. 22.4%; p=0.035), clinical pregnancy rates (34.5% vs. 20.4%; p=0.044) and ongoing pregnancy rate (32.1% vs. 18.4%; p=0.032) when compared to those who received placebo. The abortion (2.4% vs. 2.0%; p=0.929) and ectopic pregnancy rates (1.2% vs. 1.0%; p=0.976) were comparable between groups of rhCG and placebo, respectively.

**Conclusion:** Intrauterine injection of 250μg of rhCG before ET significantly improves the implantation and pregnancy rates in IVF/ICSI cycles.

Registration ID in IRCT: IRCT2012121711790N1

This article extracted from fellowship course thesis. (Masoumeh Younesi)

## Introduction

Although major advances have been achieved in assisted reproductive techniques (ART), the implantation rates have remained quite low to allow the universal use of single embryo transfer. Successful implantation needs a good quality embryo, a receptive endometrium, and an ideal embryo transfer (ET) technique (1). Currently it is estimated that about 50-75% of all pregnancies are lost due to implantation failure (2). An important feature of secretory-phase of endometrium between days 22 and 25 is the remarkable changes related to predecidual transformation of the upper two thirds of the functional is layer. 

The glands show extensive coiling and luminal secretions can be seen. The changes within the endometrium called "implantation window" become visible on days 20-24 (3). Evidence has accumulated that Interleukin-1 α (IL-1α), IL-1β, and human chorionic gonadotropin (hCG) are secreted by the blastocyst and these agents can have positive effect on the endometrium as well as endometrial receptivity (4). The understanding of the molecular and biological mechanisms of implantation could help the clinicians to overcome the shortcomings of these important steps toward fertilization (2). Implantation is a very complex process which is regulated by a large number of factors, the most important of which is hCG (5). 

Recently, it has been demonstrated that recombinant hCG (rhCG) prevents the occurrence of apoptosis in decidualizing human endometrial stromal cells (HESCs) exposed to oxidative stress (6). The direct effect of hCG on the human endometrium was studied by Licht *et al* (7). They showed that intrauterine injection of 500 IU of hCG/mL provoked significant inhibition of intrauterine insulin-like growth factor-binding protein 1 (IGFBP-1) and macrophage colony stimulating factor (M-CSF). In addition, leukemia inhibitory factor (LIF), vascular endothelial growth factor (VEGF), and matrix metalloproteinase 9 (MMP-9) were significantly stimulated. It is further demonstrated that intrauterine injection of 500 IU of hCG before ET significantly improves both implantation and pregnancy rates in in vitro fertilization/ intracytoplasmic sperm injection (IVF/ICSI) cycles (8). 

The ET technique protocols should be standardized in order to increase the outcome and fertility rate following IVF/ICSI. In a survey of 80 IVF practitioners, standardization of the ET technique was considered the most important factor influencing the success rate of IVF (9). We hypothesized that intrauterine injection of rhCG will improve the outcome of IVF/ICSI cycles. Thus the aim of this study was to determine the effects of intrauterine rhCG injection before ET on improving the implantation and pregnancy rates in IVF/ICSI cycles.

## Materials and methods

**Study Population**

This was a randomized, double blind, placebo-controlled clinical trial being performed from December 2011 to November 2012 in Reproductive Medicine Center of Mother and Child Hospital, a tertiary healthcare center affiliated with University of Medical. The study protocol was approved by both institutional review board (IRB) and ethics committee of Shiraz University of Medical Sciences. All the women provided their informed written consents. During the study period we evaluated a total number of 329 infertile women referring to our center for undergoing IVF/ICSI. 

We included those 18-40 years old women who suffered from infertility. Infertility was defined as 1 year of unprotected intercourse without conception. Semen analysis (for the partners), hormonal assay including thyroid stimulating hormone (TSH), prolactin (to rule out hypophyseal adenomas), day 3 follicle-stimulating hormone (FSH), day 3 luteinizing hormone (LH) (to rule out ovarian dysfunction such as premature ovarian failure), and Anti-mullerian hormone (AMH) were measured and hysterosalpingogram (HSG) was performed to evaluate uterine cavity. All women had normal plasma concentrations of day 3 LH, FSH and AMH; normal TSH, prolactin and HSG, and negative pregnancy tests. We excluded those who had autoimmune disorders and endocrinopathies. We also excluded those who had previous successful IVF/ICSI trials, endometriosis, azoospermia, and hydrosalpinges. 

**Intervention**

A total number of 210 patients fulfilled the study criteria and were further included in the trial. The patients were randomly assigned to two study groups using a computerized random digit generator based on their registration number in order of referral. Those assigned to first study group received 250μg (0.5^ml^) of rhCG (Ovitrelle®, Merck Serono, France) through intrauterine injection 12 minutes before ET (n=105), while the second group received intrauterine injection of normal saline (0.5^ml^) 12 minutes before ET (n=105). 

**Study protocol and assays**

All the patients underwent a complete history evaluation and physical examination by the attending gynecologist who was blinded to the study. The syringes with volume of 0.5 ml from each group were prepared by fellowship student and injected blinded by the attending gynecologist. Estradiol level was measured before oocyte pick up. The embryo transfer was performed on day 3 (cleavage stage) following oocyte pick up. The ICSI procedure was performed as previously described elsewhere (10, 11). 

All the patients were put in the lithotomy position and the cervix was visualized using a speculum. The cervical mucus was then wiped out ET was carried out using a malleable catheter (Cook Medical). The rhCG (250 μg) or normal saline was injected in the midpoint of endometrial cavity using the same catheter. Approximately 12 minutes after the intrauterine injection of rhCG or normal saline, the embryos were loaded into a new similar ET catheter and transferred. All the patients were followed and the pregnancy test was requested 2 weeks after the ET. Pregnancy was documented by transvaginal sonography, at 3 weeks of gestation after obtaining a positive pregnancy test. Main outcome measurements were implantation rate (detected by positive β-hCG) and chemical pregnancy rate (detected by positive β-hCG and sonography). 

We also recorded the abortion rate, multiple pregnancy rate, and ongoing pregnancy rate (calculated by subtracting abortion from pregnancy rate). Clinical pregnancy was defined as the observation of gestation sac with fetal echoes and pulsations on transvaginal sonography at 7^th^ week. Multiple gestational sacs are counted as one clinical pregnancy. Chemical pregnancy was defined by a rising β-hCG level in serum without the detection of a gestational sac. The abortion rate was defined as the loss of pregnancy before 20 weeks of gestation. Loss of pregnancy after 20 weeks of gestation was defined as still birth. 

**Statistical analysis**

Based on 80% power to detect 10% differences between main outcome measures of the study including pregnancy and implantation rates (p=0.05, 2-sided), 90 patients were required in each study groups. In order to compensate for non-evaluable patients and those would be lost to follow-up, we included 105 patients in each group. The statistical package for social sciences for Windows, version 16.0 (SPSS, Chicago, IL, USA) was used for data analysis. The paired *t*-test was used to compare results within groups, the independent *t*-test to compare results between the groups, and the χ^2^ test to compare proportions. Data were reported as mean±SD. A two-sided p-value less than 0.05 were considered statistically significant.

## Results

**Figure 1:**
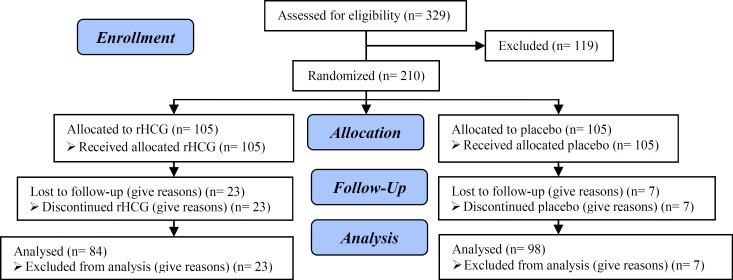
Consort flow diagram

Out of 329 patients who were assessed for eligibility, 119 were excluded and thus 210 were randomized to two study groups (each group was consisted of 105 patients). During the study, 23 patients out of intrauterine rhCG group and 7 out of placebo group were lost to follow-ups. Thus final number of patients in intrauterine rhCG and placebo groups was 84 and 98 respectively (Figure 1). The baseline characteristics of the patients were comparable between two study groups. Table I summarizes the characteristics of the patients at baseline. 

Overall, number of oocyte retrieval cycles was 240 and 262 in rhCG and placebo groups, respectively. Two study groups were also comparable regarding the IVF/ICSI cycles characteristics including number of retrieved oocytes (p=0.448), number of embryos per transfer (p=0.606) and number of 2PN embryo (p=0.601) (Table II). Patients who received intrauterine rhCG before ET had significantly higher implantation (36.9% vs. 22.4%; p=0.035), clinical pregnancy rates (34.5% vs. 20.4%; p=0.044) and ongoing pregnancy rate (32.1% vs. 18.4%; p=0.032) when compared to those who received placebo. The abortion (2.4% vs. 2.0%; p=0.929) and ectopic pregnancy rates (1.2% vs. 1.0%; p=0.976) were comparable between groups of rhCG and placebo respectively.

**Table I:** Baseline characteristics of 189 infertile patients undergoing IVF/ICSI following intrauterine injection of rhCG or placebo

		**rhCG group (n=84)**	**Placebo group (n=98)**	**p-value**
Age (years)		29.90 ± 5.225	31.29 ± 4.661	0.077
Infertility period (months)		78.7 ± 49.8	88.8 ± 59.5	0.221
Infertility type				0.549
	Primary (%)	72 (85.7%)	80 (81.6%)	
	Secondary (%)	12 (14.3%)	18 (18.4%)	
Estradiol level (ng/dL)		2220.1 ± 867.9	2060.1 ± 839.8	0.208

Student’s *t * test, χ^2^ test

**Table II:** Outcome of 182 infertile patients undergoing IVF/ICSI following intrauterine injection of rhCG or placebo

	**rhCG group (n=84)**	**Placebo group (n=98)**	**p-value**
No. of oocyte retrieval cycles	240	262	
No. of retrieved oocytes	9.8 ± 4.7	10.4 ± 6.4	0.448
No. embryos per transfer	6.1 ± 3.5	5.7 ± 4.1	0.606
No. of 2PN embryo	5.8 ± 4.5	5.4 ± 4.6	0.601
Implantation rate (%)	31 (36.9%)	22 (22.4%)	0.035
Clinical pregnancy rate (%)	29 (34.5%)	20 (20.4%)	0.044
Abortion rate (%)	2 (2.4%)	2 (2.0%)	0.929
Ectopic pregnancy rate (%)	1 (1.2%)	1 (1.0%)	0.976
Ongoing pregnancy rate (%)	27 (32.1%)	18 (18.4%)	0.032

Student’s *t * test, χ^2^ test

## Discussion

In this randomized clinical trial we tried to investigate the effects of intrauterine rhCG injection before ET on the outcome of the IVF/ICSI cylces. We found that intrauterine rhCG injection before ET significantly improves the implantation, clinical and ongoing pregnancy rates. The abortion and ectopic pregnancy rates were also comparable between rhCG and placebo groups. The results indicate that the injection of 250µg rhCG before ET would improve the outcome of IVF/ICSI. The effects of hCG on implantation and outcome of ARTs have been previously investigated (8, 12-17). 

Successful embryonic implantation requires an appropriate communication network between the embryo and its near environment within the implantation site. The underlying mechanism of improvement of implantation and pregnancy outcome following intrauterine injection of rhCG could be investigated through the pharmacokinetics of hCG. It was shown by Bourdiec and co-workers that in response to hCG administration, the endometrial cell proliferation and migration will be increased (12). hCG also increases IL1R1 and significantly downregulates IL1R2 which in turn increases the secretion of IL8. Which embryo implantation enhances. In other words, these findings show that hCG may target endothelial cells to directly stimulate angiogenesis and embryonic growth (12). It has been previously demonstrated that hCG is a trophoblast marker which is secreted by blastocyst before and after implantation (13-15).

Despite all these in vitro studies, clinical studies are scarce (12-17). Mansour and colleagues clearly demonstrated that intrauterine injection of 500 IU of hCG before ET significantly improved the implantation and pregnancy rates in IVF/ICSI cycles (8). These authors used urinary hCG (uhCG) in their trial. Several reports have indicated that the rhCG is more efficient than uhCG during IVF (18, 19). rhCG has also stronger effects on human endometrium compared to uhCG by prevention of apoptosis in decidualizing HESCs exposed to oxidative stress (6). Thus we used rhCG in our trial. Our results are consistent with that of Mansour *et al* demonstrating that the intrauterine injection of rhCG before ET would improve the outcome of IVF/ICSI cycles (8). 

To the best of our knowledge this is the first study demonstrating the positive effects of intrauterine rhCG injection on the outcome of IVF/ICSI. hCG encompasses several roles on the endometrium. It is well established that the serum level of hCG positively correlates with the level of trophoblast tolerance as well as the number of uterine natural killer cells, which play a major role in the establishment of pregnancy (20). Local immune tolerance is also induced by the hCG through cellular system apoptosis (21). 

Decidual immunity determined by TH1 (T Helper 1)/TH2 (T Helper 2) balance and the complement C_3_ and C_4_ factors which are regulated via hCG. The gestational transient tolerance is mediated by attracting regulatory T cells at the fetal-maternal interface by hCG (22). hCG also induces endometrial angiogenesis at the site of implantation by up regulating the hCG receptors (12). Embryos have been shown to secrete hCG which may influence the endometrial receptivity and, consequently, the receptive endometrium will respond by producing LIF and colony-stimulating factor-1 (CSF-1) (4). It has also been proved that hCG plays a critical role in proliferation of myometrium smooth muscle cells (23). According to another study, the administration of hCG (500 IU/mL) stimulated significant inhibition of intrauterine IGF-binding protein-1 (IGFBP-1) and M-CSF. 

On the other hand, LIF (a cytokine required for implantation), VEGF (a proangiogenic growth factor), and MMP-9 (a regulator for tissue remodeling) were significantly stimulated (7). We note some limitations to our study. First, the number of patients who were lost to follow-up was approximately high. However as we have included more patients, the final power of the study was higher than 80%. We injected 250μg of rhCG although previous report had shown that 500 IU of hCG is effective in improving the pregnancy rate. However we achieved the same results i.e. increased pregnancy and implantation rates following IVF/ICSI. Future trials are required to investigate the effects of rhCG on the outcome of IVF/ICSI. 

## Conclusion

In conclusion, intrauterine injection of 250μg of rhCG before ET significantly improves the implantation and pregnancy rates in IVF/ICSI. These favorable effects of rhCG are secondary to its angiogenesis and immune tolerance inducing potentials.
